# A Case of Ruminative Hypomania Induced by High Dose Venlafaxine

**DOI:** 10.1192/j.eurpsy.2022.1051

**Published:** 2022-09-01

**Authors:** E. Yıldızhan, M. Ünlü Çilesiz, E. Ekici, N. Tomruk

**Affiliations:** Bakirkoy Prof. Dr. Mazhar Osman Mental Health and Neurological Diseases Training and Research Hospital, Psychiatry 14, İstanbul, Turkey

**Keywords:** venlafaxine, BIPOLAR, obsession, Mixed features

## Abstract

**Introduction:**

Obsessive phenomena, when present, are usually seen in the depressive phase of bipolar disorder.

**Objectives:**

The peculiar case with aggravation in ruminative and obsessive thinking with simultaneous hypomania may widen our understanding of the phenomenology of antidepressant induced hypomanic symptoms.

**Methods:**

We present a case of ruminative hypomania induced by high dose venlafaxine. Young Mania Rating Scale (YMRS), Hamilton Depression Rating Scale (HAM-D) and Yale Brown Obsessive Compulsive Scale (YBOCS) were used for symptom ratings.

**Results:**

The patient was 30 years old and she had treatment history of depression for 3 months. She had two consecutive suicide attempts with drugs in the week before she was hospitalized for suicidal risk. She was using venlafaxine 300 mg/day and olanzapin 2,5 mg/day; continuous ruminative thinking about the past and imaginary sexual affairs with former friends were apparent with an unremitting pattern, leading to intense psychomotor agitation and suicide attempts. Irritable mood, and increased energy was observed with continuous ruminations. She was diagnosed with bipolar-II-disorder, with mixed features and anxious distress (YMRS:17, HAM-D:22, YBOCS:34). After discontinuing venlafaxine and starting anti-manic treatment with haloperidol 10 mg/day in the first week, both affective symptoms and ruminations were improved (YMRS:2, HAM-D:4, YBOCS:8). Aripiprazol 20 mg/day and quetiapine 100 mg/day which were given for continuation treatment were also effective for preserving full remission.

**Conclusions:**

When prescribing high dose venlafaxine for treatment resistant depression, it should be remembered that this may induce hypomanic symptoms and prominent ruminative thinking which can be ameliorated with anti-manic treatment.

**Disclosure:**

No significant relationships.

